# Transcorneal electrical stimulation: impact on healthcare and future potential

**DOI:** 10.3389/fcell.2025.1569759

**Published:** 2025-05-16

**Authors:** Takeshi Morimoto

**Affiliations:** Department of Advanced Visual Neuroscience, The University of Osaka Graduate School of Medicine, Suita, Osaka, Japan

**Keywords:** transcorneal electrical stimulation, neuroprotection, photoreceptor, retinal ganglion cell, neuromodulation

## Abstract

Transcorneal electrical stimulation (TES), a noninvasive therapeutic technique, has gained attention for its potential to treat retinal and optic nerve diseases. TES involves applying weak electrical currents via electrodes on the cornea to stimulate retinal ganglion cells (RGCs) without causing activation of photoreceptors, inducing phosphenes, and enabling the evaluation of inner retinal function. This is valuable for assessing residual retinal activity in patients with photoreceptor or RGC degeneration. Furthermore, TES has shown significant neuroprotective effects on RGCs and photoreceptors through mechanisms involving the upregulation of neurotrophic factors (e.g., insulin-like growth factor 1, brain-derived neurotrophic factor, and ciliary neurotrophic factor), reduction of inflammatory responses, and enhanced ocular blood flow. These findings are supported by extensive animal studies, showing its efficacy in mitigating retinal degeneration and optic nerve damage while promoting axonal regeneration. Clinically, TES has shown potential in improving visual function in diseases such as RP, optic neuropathies, and ischemic retinal conditions; however long-term benefits remain a challenge. Randomized controlled trials have indicated the safety and modest therapeutic effects of TES, suggesting its potential as an adjunct treatment for visual impairments. Moreover, TES may extend beyond ophthalmology into neurology. Because the retina is anatomically connected to the brain, TES can influence brain regions such as the visual cortex and hippocampus. Preliminary research proposes its potential for modulating brain, such as those with retinitis pigmentosa (RP). TES has demonstrated significant neuroprotective effects in networks, cognition, and emotional pathways, offering hope for treating neurodegenerative diseases such as Alzheimer’s and Parkinson’s disease. In summary, TES represents a versatile and promising therapy for retinal and neurological disorders, and ongoing advancements will likely expand its applications in clinical practice. Further studies are warranted to optimize its parameters, enhance its efficacy, and explore its full therapeutic potential.

## 1 Introduction

Electrical stimulation (ES) is a promising therapeutic tool for treating various neurological disorders. Multiple studies have demonstrated significant beneficial effects of ES with optimal safety and feasibility.

Vagus nerve stimulation (VNS) is clinically applied for the treatment of epilepsy, depression, cluster headache, and migraine (Cheng et al., 2022; Austelle et al., 2024). Deep brain stimulation has been applied in clinical practice for over 25 years and is well-established as an effective treatment for Parkinson’s disease (PD), dystonia, and Tourette syndrome (Ranjan et al., 2024).

Transcranial electrical stimulation (tES) has also been extensively investigated to alter brain function noninvasively by applying current to electrodes on the scalp. tES can induce changes in synaptic excitability and is promising for enhancing recovery in patients with stroke (Motolese et al., 2022). In addition, tES is clinically applied for the treatment of Alzheimer’s disease (AD) (Pilloni et al., 2022), Cerebral vasospasm and delayed cerebral ischemia after aneurysmal subarachnoid hemorrhage are the leading causes of morbidity and mortality after aneurysmal subarachnoid hemorrhage (Budohoski et al., 2014). Several types of ES have been tested for the treatment of cerebral vasospasms and delayed cerebral ischemia, including trigeminal/vagus/facial nerve stimulation, sphenopalatine ganglion and spinal cord stimulation, tES, transcutaneous electrical neurostimulation, and electroacupuncture (Powell et al., 2022).

For the retina and optic nerve (ON), transcorneal ES (TES) modulates retinal neurons to evoke light sensations, commonly referred to as “phosphene.” This phenomenon has been utilized to evaluate the residual retinal function in individuals with visual impairments. Furthermore, owing to the neuroprotective effects of TES on injured retinal ganglion cells (RGCs) *in vivo* (Morimoto et al., 2005), basic and clinical research on TES and related ES methods have significantly progressed over the past two decades. These advancements have established ES as a promising treatment approach for ON and retinal diseases (Morimoto, 2012; Pardue et al., 2014; Tao et al., 2016; Liu et al., 2021; Li et al., 2024).

This review explores the fundamental and clinical studies conducted on TES to date and its potential future applications.

## 2 History of transcorneal electrical stimulation

ES of the eye can induce a light sensation known as “phosphine,” a phenomenon that later led to the development of retinal prostheses aimed at restoring vision in blind patients with advanced retinitis pigmentosa (RP).

**FIGURE 1:**
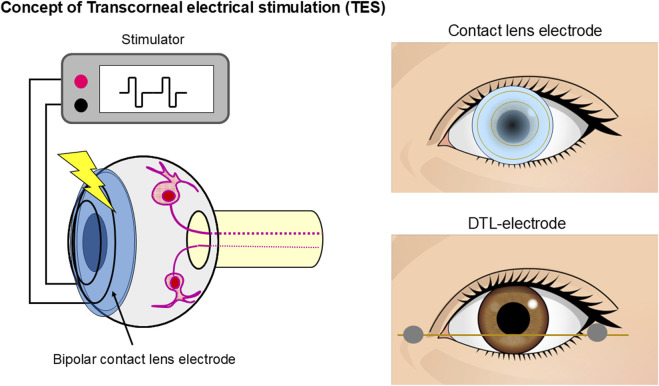
Schematic representation of TES. A bipolar contact lens electrode with an inner and outer ring in the form of a contact lens or DTL-electrode was placed on the participant’s cornea. Electric current pulses activate the retinal neurons.

TES is used to stimulate the retina and evoke phosphenes. The procedure involves placing a bipolar contact lens electrode with an inner and outer ring in the form of a contact lens, such as an electroretinogram (ERG) electrode, or DTL-electrode on the patient’s cornea (Figure 1). To stimulate the retina, a weak electric current is then applied through the electrodes. Numerous studies have investigated ES-induced phosphenes. Early research on electrically induced phosphenes primarily focused on psychophysical studies (Motokawa et al., 1951; Brindley, 1960).

Subsequently, Jarvik and Kopp (1967) developed a more convenient and less invasive method known as TES. TES was then employed in electrophysiological studies to investigate the relationship between ES and phosphene perception. Potts et al. (1968) were the first to report an “electrically evoked potentials (EER)” induced by TES. They found that the latency of EER was shorter than that of the visually evoked potentials, indicating that TES can evoke visual perception even without photoreceptor involvement (Potts et al., 1968; Potts and Inoue, 1969; 1970). Shimazu et al. (1996) and Shimazu et al. (1999) also reported similar findings using experiments with cats.

Subsequently, several human experiments have evaluated the characteristics of EERs in healthy participants (Miyake et al., 1980a; Dorfman et al., 1987; Takei et al., 1993) and patients with various retinal and ON diseases (Miyake et al., 1980b; Miyake et al., 1980c; Dorfman et al., 1987). Studies have reported that the EERs remained nearly normal in patients with functional disorders of the rod or cone visual pathways (Miyake et al., 1980b), whereas they were reduced in patients with central retinal artery occlusion or ON diseases (Miyake et al., 1980c). Despite these findings, basic research on TES made no significant progress, nor did it lead to clinical applications, and studies on TES came to a standstill for some time.

## 3 TES to assess inner retinal function

The advancement in retinal prostheses and regenerative medicine renders it possible to restore the vision of patients with blindness and retinal degenerative diseases such as RP. For the successful restoration of vision through such treatment, the function of the inner retinal neurons must remain intact.

As the prospect of clinical applications transitions into reality, robust and sophisticated methodologies are increasingly needed to assess residual inner retinal function in patients with blindness (Table 1), particularly those being considered candidates for these transformative treatments.

**TABLE 1:** Summary of clinical studies on the evaluation of inner retinal function by TES and similar ES.

Study	Subject	Electrode	Parameters	Evaluation method
Potts et al. (1968)	Healthy (n = 8)	Contact lens electrode	Monophasic 0.3–2.3 mA, 5.0–50 ms duration	EER
Potts and Inoue. (1969)	RP (n = 4)	Contact lens electrode	Monophasic 2.0 mA, 5.0 ms duration	EER
Miyake et al. (1980a)	Healthy (n = 50)	Contact lens electrode	Monophasic, 0.1–2.0 mA, 5.0 ms duration, 1.98 Hz	EER
Miyake et al. (1980b)	IRD (n = 5)	Contact lens electrode	Monophasic, 0.1–2.0 mA, 5.0 ms duration, 1.98 Hz	EER
Miyake et al. (1980c)	CRAO (n = 8)	Contact lens electrode	Monophasic, 0.1–2.0 mA, 5.0 ms duration, 1.98 Hz	EER
Dorfman et al. (1987)	Ocular trauma (n = 17), Healthy (n = 4)	Contact lens electrode	Monophasic, 2.0–20 mA, 1.0 ms duration, 1.0 Hz	EER
Takei et al. (1993)	Healthy (n = 23), MH (n = 6), RAO (n = 3)	Contact lens electrode	Monophasic 0.3–2.0 mA 5 ms duration, 1.85 Hz	EER
Morimoto et al. (2006)	IRD (n = 20), Healty (n = 8)	Contact lens electrode	Biphasic, 0.05–2.0 mA, 10 ms/phase, 20 Hz 1.0 s	Subjective phosphene and pupillary reflex
Gekeler et al. (2006)	Healthy (n = 17), POAG (n = 9), RP (n = 14), Amblyopia (n = 3), Homonymous visual field loss (n = 4).	DTL electrode	Monophasic, 0–4.0 mA, 0.05–50.0 ms duration, 0.67 Hz,	Subjective phosphene
Fujikado et al. (2007)	Healty (n = 6), RP (n = 2)	Monopolar scleral electrode	Biphasic, 1.0–1.5 mA, 1.0 ms/phase+interpulse 1.0 ms , 20 Hz, 20 pulses or 0.5–4.0 ms/phase+interpulse 1.0 ms, 50 Hz, 20 pulses	Subjective phosphene and pupillary reflex
Huang et al. (2009)	RP (n = 17), Healthy (n = 15)	Contact lens electrode	Biphasic, 0.025–1.0 mA, 5, 7.5, 10 ms /phase, 20 Hz, 2 s	Subjective phosphene, OCT
Naycheva et al. (2012)	Healthy (n = 20), RP (n = 30) ,STG(n = 14), RAO (n = 20), NAION (n = 16), POAG (n = 17)	DTL electrode	Biphasic, 0–10 mA, 1–100 Hz, 5 ms/phase	Subjective phosphene
Kelbsch et al. (2017)	RP (n = 40), Healthy(n = 40)	DTL electrode	Biphasic, 0–1.2 mA, 10 ms/phase, 20 Hz	Subjective phosphene and pupillary reflex
Kelbsch et al. (2018)	Healthy (n = 14)	DTL electrode	Sinusoidal, 0.01, 0.02,0.05 mA, 10 or 20 Hz, envelope frequency 1.2 Hz	Subjective phosphene and pupillary reflex

CRAO, central retinal arterial occlusion; DTL, Dawson–Trick–Litzkow; EER, electrically evoked response; ES, electrical stimulation; IRD, Inherited retinal degeneration; MH, macular hole; NAION, nonarteritic anterior ischemic optic neuropathy;OCT, optical coherence tomography; ON, optic neuropathy; POAG, primary open-angle glaucoma; RAO, retinal arterial occlusion; RP, retinitis pigmentosa; STG, Stargardt disease; TES, transcorneal electrical stimulation.

In particular, patients eligible for treatment often suffer from degeneration-induced photoreceptor loss, which makes it impossible to evaluate inner retinal function using conventional ophthalmic tests such as visual acuity (VA) tests, visual field (VF) tests, ERG, or VEP. The structural evaluation of the inner retinal layers using optical coherence tomography (OCT) is currently the sole method for assessing the inner retinal layers (Chader et al., 2009).

TES is considered an effective method for evaluating inner retinal functions because it can stimulate RGCs without activating photoreceptors (Potts and Inoue, 1970; Miyake et al., 1980b; Shimazu et al., 1999). This has brought TES back into the spotlight, leading to a resurgence of its research starting in the 2000s. A method combining TES-induced phosphenes and pupil responses to evaluate inner retinal function subjectively and objectively in healthy individuals and patients with inherited retinal degeneration has been reported (Morimoto et al., 2006; Kelbsch et al., 2017; Kelbsch et al., 2018).

Methods have been developed to evaluate the function of the inner retinal layers by analyzing the characteristics of ES-induced phosphenes, such as their position, size, shape, brightness, and color (Gekeler et al., 2006; Fujikado et al., 2007; Naycheva et al., 2012). Furthermore, a combined approach integrating OCT for retinal structural assessment with the evaluation of phosphenes was also proposed (Huang et al., 2009).

Given the rapidity, safety, and reliability of phosphene-based evaluation of inner retinal function using TES, this method shows great potential for assessing inner retinal layer function in patients with blindness and inherited retinal degeneration and could become a standard diagnostic test.

In the future, with the wide adoption of regenerative medicine, retinal prostheses, and optogenetic therapies, TES is expected to play an increasingly crucial role in assessing the functionality of the inner retinal layers in patients who have lost photoreceptors.

## 4 Neuroprotective effects of TES on the retinal neurons

ES can dose-dependently modulate the survival rates of isolated central nervous system (CNS) neurons *in vitro* (Kaplan et al., 1988). Many studies have investigated the neuroprotective effects of ES on injured neurons *in vivo*. Within the auditory system, the survival of spiral ganglion cells (SGCs) is a key factor that influences the performance of cochlear implants. Enhancing SGC survival is anticipated to improve sensitivity and enhance auditory discrimination. Chronic ES supported SGC survival that would otherwise degenerate following exposure to ototoxic drugs *in vivo* (Lousteau, 1987; Hartshorn et al., 1991).

Similarly, in the visual system, brief ES using monophasic pulses on the transected ON increased RGC survival in rats, demonstrating the neuroprotective effect of ES on the ON (Morimoto et al., 2002). Furthermore, the extent of this survival-promoting effect was dependent on the ES parameters (Okazaki et al., 2008).

Direct ES of the ON has demonstrated a neuroprotective effect; however, its highly invasive nature makes its clinical application challenging. Therefore, this study focused on TES, a less invasive and safer stimulation method than ON stimulation, which is also used to evaluate the function of inner retinal layers. This study revealed that TES exerts a neuroprotective effect on RGCs, similar to that of direct ON stimulation. TES increased RGC survival after ON transection in rats by upregulating endogenous IGF-1 (Morimoto et al., 2005). The survival-promoting effect of TES was dependent on ES parameters (Morimoto et al., 2010). TES was also neuroprotective for axons in crushed ONs (Miyake et al., 2007) and enhanced the axonal regeneration of RGCs through the activation of the IGF-1 pathway in the rat ON crush model (Tagami et al., 2009).

Moreover, TES exerts neuroprotective effects on photoreceptors. In animals with inherited photoreceptor degeneration, TES enhanced photoreceptor survival in Royal College of Surgeons rats (Morimoto et al., 2007; Gonzalez Calle et al., 2023), P347L transgenic rabbits (Morimoto et al., 2012), P23H rats (Rahmani et al., 2013), N-methyl-N-nitrosourea-administered mice (Tao et al., 2016), rd 10 mice (Liu et al., 2022), and phototoxic rats (Ni et al., 2009), rhodopsin knockout mice (Enayati et al., 2024). TES also exerted neuroprotective effects on ischemic damaged retinas *in vivo* (Wang et al., 2011). TES provided RGC axon protection and led to a reduction in inflammatory cells in a mouse glaucoma model (Jassim et al., 2021).

The results of numerous animal experiments have demonstrated the neuroprotective effects of TES in the eyes of patients with retinal degenerative diseases and ON disorders (Table 2).

**TABLE 2:** Summary of preclinical studies of TES and similar ES.

Study		Animal	Model	Electrode	Parameters	Effect
Morimoto et al. (2002)		Wistar rats (ON transection)	TON	Optic nerve monopolar electrodes	Monophasic 0.02–0.07 mA, 0.05 ms duration, 20 Hz 2 h, once	RGC survival
Morimoto et al. (2005)		Wistar rats (ON transection)	TON	Contact lens electrode	Biphasic 0.1 mA, 0.5–3.0 ms/phase, 20 Hz, 1.0 h, once	RGC survival
Morimoto et al. (2007)		RCS rats	RP	Contact lens electrode	Biphasic 0.05–0.1 mA, 1.0 ms/phase, 20 Hz, 1 h, once a week for 2–6 wk	PR survival
Miyake et al. (2007)		Wistar rats (ON crush)	TON	Contact lens electrode	Biphasic 0.5 mA, 0.05 ms/phase, 20 Hz, 6 h, once	RGC survival
Okazaki et al. (2008)		Wistar rats (ON transection)	TON	Optic nerve monopolar electrodes	Monophasic 0.05 mA, 0.05 ms duration, 10–50 Hz 10–120 min, once	RGC survival
Tagami et al. (2009)		Wistar rats (ON crush)	TON	Contact lens electrode	Biphasic 0.1 mA, 1 ms/phase, 20 Hz, 1 h, 1,2,4,12 times for 12 d	Axonal regeneration of RGCs
Ni et al. (2009)		SD rats (light-induced)	RP	Contact lens electrode	Pre: biphasic 0.1–0.5 mA, 3 ms/phase, 20–100 Hz, 1 hr, once Post: biphasic 0.2-0.3 mA, 3 ms/phase, 20 Hz, 1 hr, every 3 d for 1-2 wk	PR survival
Morimoto et al. (2010)		Wistar rats (ON transection)	TON	Contact lens electrode	Biphasic 0.1 mA, 1 ms/phase, 20 Hz, 1 h, once	RGC survival
Wang et al. (2011)		SD rats(high IOP)	ION	Contact lens electrode	Biphasic 0.3 mA, 3 ms/phase, 20 Hz, 1 h, every 2 d for 2 wk	RGC survival
Morimoto et al. (2012)		P347L transgenic rabbits	RP	Contact lens electrode	Biphasic 0.7 mA, 10 ms/phase, 20 Hz, 1 h, once a week for 6 wk	PR survival
Rahmani et al. (2013)		P23H rats	RP	Sintered pellet electrodes, cornea & mouth	Sinusoidal, 4.7 mA, 5 Hz, 30 min, twice a week for 12 wks	PR function (ERG)
Tao et al. (2016)		C57/BL mice(MNU treated)	RP	Contact lens electrode	Biphasic 0.1–0.2 mA, 20 Hz, 1 h, three times for a week	PR survival
Jassim et al. (2021)		DBA/2J (D2) mice	Glaucoma	Contact lens electrode	Biphasic 0.1 mA, 1 ms/phase, 20 Hz, 10 min, every 3 d for 8 wk	RGC survival
Liu et al. (2022)		rd10 mice	RP	Sclera electrode	Biphasic 0.05–0.1 mA, 2.5 ms/phase +interpulse 1 ms, 20 Hz, 1 hr, 3 or 5 times for 5 d	PR survival
Gonzalez Calle et al. (2023)		RCS rats	RP	Cornea ring electrode	Biphasic 0.2–0.1 mA, 10 ms/phase, 6 Hz, 2 hr, once a week, 6 times	PR survival
Enayati et al. (2024)		rhodopsin knockout mice	RP	Skin electrodes (upper and lower eye lids)	Monophasic, rectangular (0.1 mA, 2–200 Hz, 40 s/cycle, 160 s) + ramp waveform (0.1 mA, 20 Hz, 160 s), 5 d x 2 times	Improvement in retinal function and visual behavior

ERG, electroretinogram; ION, ischemic optic neuropathy; IOP, intraocular pressure; MNU, N-methyl-N-nitrosourea; ON, optic nerve; PR, photoreceptor; RCS, Royal College of Surgeon; RGC, retinal ganglion cell; RP, retinitis pigmentosa; TON, traumatic optic neuropathy.

## 5 Mechanism of the neuroprotective effects of TES on the retina and ON

The mechanism underlying the neuroprotective effects of ES has been extensively studied over time. As regards the neuroprotective and axonal outgrowth-promoting effects of ES, ES-induced depolarization via the activation of voltage-dependent Ca^2+^ channels is crucial.

Brief periods of ES applied to cultured *Xenopus* spinal neurons significantly increased intracellular Ca^2+^ and cAMP levels, which, in turn, play a crucial role in promoting the extension of growth cones (Ming et al., 2001). Various neurotrophic factors are reportedly induced by ES applied to RGCs and/or Müller cells, exerting neuroprotective effects *in vitro*.

RGC stimulation by ES from a silicon chip enhanced their survival and axonal growth in response to brain-derived neurotrophic factor (BDNF) *in vitro* (Goldberg et al., 2002).

Brief ES of cultured Müller cells increased the gene expression of IGF-1, BDNF, basic fibroblast growth factor (bFGF) (Sato et al., 2008a; Sato et al., 2008b; Sato et al., 2008c), and ciliary neurotrophic factor (CNTF) (Enyati et al., 2020) by activating L-type voltage-dependent Ca^2+^channels.

The neuroprotective effects of TES are considered to involve various neurotrophic and neuroprotective factors that are expressed within the retina in response to TES *in vivo*. TES enhanced retinal neuron survival by increasing endogenous neurotrophic factors, namely, IGF-1 (Morimoto et al., 2005), BDNF and CNTF (Ni et al., 2009; Tao et al., 2016), and bFGF (Yu et al., 2020). These neurotrophic factors increased significantly in Müller cells, which play a significant role in TES-induced neuroprotection.

Other neuroprotective factors were reported to be related to the neuroprotective effects of TES. Bcl-2 was upregulated, whereas Bax was downregulated (Ni et al., 2009; Tao et al., 2016), and the tumor necrosis factor superfamily was upregulated in the retina after TES (Willmann et al., 2011). DNA methylation changes with therapeutic effects were also induced by TES (Tew et al., 2024).

TES also affects the immune system. TES decreased the number of Iba-1-positive microglial cells, reduced interleukin-6 (IL-6) and COX-2 expression and NF-κB phosphorylation, and increased IL-10 levels (Fu et al., 2018). Microglial inhibition by TES was observed in genetic secondary glaucoma mouse model (Jassim et al., 2021).

Other effects of TES are thought to be associated with increased ocular blood flow. Numerous studies have investigated the potential relationship between TES and retinal blood flow, and growing evidence indicates a significant link. For instance, measurements of TES-induced retinal intrinsic reflective changes in cat eyes have revealed vascular changes caused by the activation of retinal neurons (Mihashi et al., 2011; Morimoto et al., 2014). Similarly, studies assessing blood flow in human eyes have demonstrated that TES increases chorioretinal blood flow in both healthy individuals (Kurimoto et al., 2010) and patients with RP (Bittner et al., 2018a). Furthermore, the TES-induced increase in retinal blood flow involves neurovascular coupling (NVC) (Su et al., 2020).

NVC is a phenomenon in which neurons, glial cells, and blood vessels in the CNS work together. When neurons become active, the blood flow in the corresponding region increases to meet the energy demands of the active neurons by delivering oxygen and glucose. NVC is essential for supporting RGC metabolism and survival (Haider et al., 2022). Many patients with glaucoma suffer from vascular deficits, including reduced blood flow, impaired autoregulation, NVC dysfunction, and breakdown of the blood–retina and blood–brain barriers (Alarcon-Martinez et al., 2023).

Based on the above findings, increased blood flow is inferred to exert a neuroprotective effect on the retina. The TES-induced increase in ocular and retinal blood flow may contribute to this neuroprotective effect.

TES also affects neuronal activity in the visual pathway and ameliorates retinal-genicular- cortical function in diseases involving the visual system (Castoldi et al., 2025).

**FIGURE 2:**
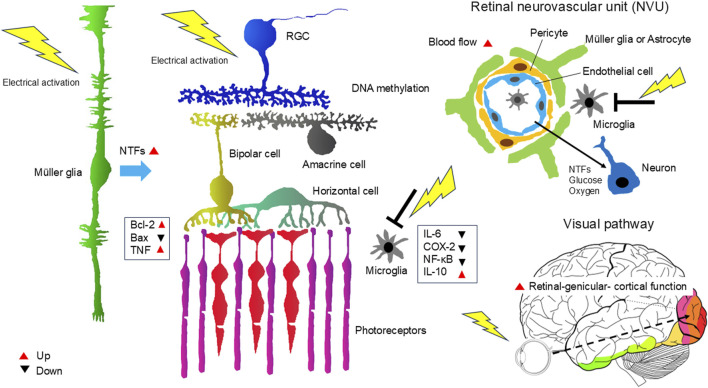
Mechanism of the neuroprotective effects of TES on the retina and ON. TES triggers various responses that act together to promote neuronal survival and improve neuronal function. These include the production of neurotrophic factors via Müller cells, DNA methylation, modulation of the immune system (e.g., suppression of macrophage activity), and an increase in ocular and retinal blood flow, and amelioration of retinal-genicular- cortical function in ocular diseases involving the visual system. NTFs, neurotrophic factors.

In summary, TES is thought to exert neuroprotective effects on RGCs and photoreceptors through various mechanisms. These include the production of neurotrophic factors via Müller cells, DNA methylation, modulation of the immune system (e.g., suppression of macrophage activity), and an increase in ocular and retinal blood flow, and amelioration of retinal-genicular- cortical function in ocular diseases involving the visual system (Figure 2).

## 6 Clinical application of TES for various retinal and ON diseases

Numerous clinical studies have examined TES and similar ES therapies for various retinal and ON diseases (Table 3). Initial TES was performed for nonarteritic anterior ischemic optic neuropathy and traumatic optic neuropathy, and some patients reported improvements in VA and VFs (Fujikado et al., 2006). Since then, TES has been applied to diseases, including retinal artery occlusion (Inomata et al., 2007; Oono et al., 2011; Naycheva et al., 2013), Best vitelliform macular dystrophy (Ozeki et al., 2013), ON atrophy (Gall et al., 2010), and glaucoma (Ota et al., 2018). Despite the modest therapeutic effects, positive outcomes were observed, leading to the application of this treatment approach to various ocular diseases.

**TABLE 3:** Summary of clinical studies of TES and similar ES therapies.

Study	Subject	Design	Type	Electrode	Parameters	Outcome
Fujikado et al. (2006)	ION (n = 3), TON (n = 5)	Case series	TES	Contact lens electrode	Biphasic 0.3-2 mA, 10 ms/phase, 20 Hz, 30 min, once	Improvement in VA and VF
Inomata et al. (2007)	CRAO (n = 2), BRAO (n = 1)	Case series	TES	Contact lens electrode	Biphasic 1.1 mA, 10 ms/phase, 20 Hz, 30 min, once a month for 3 mo	Improvement in VA and VF
Gall et al. (2010)	TON (n = 1)	Case report	rtACS	Skin electrode (upper eye lid)	Biphasic, current bursts, <0.6 mA, 10–30 Hz, 30–40-min for 10 d	Increase of detection ability and mean perimetric threshold
Schatz et al. (2011)	RP (n = 24)	Prospective, randomized, partially blinded study	TES	DTL electrode	Biphasic intensity 66% or 150% of EPT, 5 ms/phase, 20 Hz, 30 min, once a week for 6 wk	Improvement in VF at 150% of EPT
Oono et al. (2011)	BRAO (n = 5)	Case series	TES	Contact lens electrode	Biphasic 0.5–0.9 mA, 10 ms/phase, 20 Hz, 0.5 hour, once	Improvement in visual function (mERG, HFA)
Gall et al. (2011)	OND (n = 24), OND (placebo, n = 18)	Prospective, randomized, sham controlled study	rtACS	Skin electrode (upper eye lid)	Biphasic, current bursts, <1.0 mA, 5-20 Hz, 20-40 min daily for 10 d	Increase of detection ability
Sabel et al. (2011)	OND (n = 12), OND (placebo, n = 10)	Prospective, randomized, double-blind, placebo controlled study	rtACS	Skin electrode (upper eye lid)	Biphasic, current bursts, <1.0 mA, 5–20 Hz, 15 min daily for 10 d	Improvement of central visual field
Fedorov et al. (2011)	OND (n = 446)	Open-label, clinical observational study	rtACS	Skin electrode (upper eye lid)	Biphasic, current bursts, <1.0 mA, 5–20 Hz, 25–40 min daily for 10 d	Improvement in VF and VA
Naycheva et al. (2013)	CRAO (n = 10, sham n = 2), BRAO (sham n = 1)	Prospective, randomized, sham-controlled study	TES	DTL electrode	Biphasic intensity 66% or 150% of EPT, 5.0 ms/phase, 20 Hz, 30 min, once a week for 6 wk	Improvement in ERG response (a-wave) at 150% of EPT
Ozeki et al. (2013)	BVMD (n = 1)	Case report	TES	Contact lens electrode	Biphasic 0.17-0.25 mA, 10 ms/phase, 20 Hz, 30 min, 4 times	Improvement in VA
Gall et al. (2016)	OND (n = 45), OND (sham, n = 37)	Multicenter, prospective, randomized, double-blind, sham-controlled study	rtACS	Skin electrode (upper eye lid)	Biphasic, current bursts, 125% of EPT, 8–25 Hz, 50 min 10 d within 2 weeks	Improvement in VF
Schatz et al. (2017)	RP (n = 32), RP (sham, n = 20)	Prospective, randomized, partially masked study	TES	DTL electrode	Biphasic intensity 150% or 200% of EPT, 5 ms/phase, 20 Hz, 30 min per week for 52 consecutive wk	Improvement of retinal function (scotopic b-wave) at 200% of EPT
Wagner et al. (2017)	RP (n = 7)	Prospective open-label observational study	TES	DTL electrode	Biphasic intensity 150% of EPT or 1.0 mA, 5 ms/phase, 20 Hz, 30 min, once a week for 6 mo, 24 sessions	No improvement in visual function compared to the control eyes
Ota et al. (2018)	POAG (n = 3), NTG (n = 2)	Case series	TES	DTL electrode	Biphasic 0.3-0.5 mA, 10 ms/phase, 20 Hz, 30 min, every 3 mo for 11–68 mo	Improvement in VF (POAG)
Bittner and Seger (2018b)	RP (n = 7)	Prospective, randomized, controlled study	TES	DTL electrode	Biphasic 0.75 mA, 5 ms/phase, 20 Hz, 30 min, once a week for 6 wk	Prevention of slowly diminishing vision (ETDRS VA, GVF, qCSF)
Miura et al. (2019)	RP (n = 10)	Prospective, non-randomized, open-label, uncontrolled study	TdES	Skin electrode (lower eye lid)	Biphasic 1.0 mA, 10 ms/phase, 20 Hz, 30 min, every 2 weeks for 6 sessions	Improvement of ETDRS BCVA and HFA VF
Jolly et al. (2020)	RP (n = 105)	Single-arm open label interventional safety study	TES	DTL electrode	Biphasic < 1.0 mA, 5 ms/phase, 20 Hz, 30 min, once a week for 6 mo	Transient dry eye symptoms, no serious adverse events, no improvement in visual function
Demir et al. (2022)	RP (n = 15)	Prospective, randomized, controlled study	TES	DTL electrode	Biphasic 200% of EPT, 2 ms/phase, 20 Hz, 30 min, once a week for 12 wk	Improvement in BCVA, color vision, mERG(ring1)
Sinim Kahraman and Oner (2020)	RP (n = 101)	Prospective, randomized, controlled study	TES	DTL electrode	Biphasic 150% of EPT, 5 ms/phase, 20 Hz, 30 min, once a week for 8 wk	Improvement in VA or VF at 1 mo after TES
Kurimoto et al. (2020)	LHON (n = 10)	Prospective, non-randomized, open-label, uncontrolled study	TdES	Skin electrode(lower eye lid)	Biphasic 1.0 mA, 10 ms/phase, 20 Hz, 30 min, every 2 wk for 6 sessions	Improvement in VA
Dizdar Yigit et al. (2022)	RP (n = 15)	Prospective, randomized, fellow-eye–controlled study	TES	DTL electrode	Biphasic 200% of EPT, 5 ms/phase, 20 Hz, 30 min, once a week for 6 mo	Stabilization of retinal function (mERG)
Stett et al. (2023)	RP (n = 31), RP (sham, n = 20)	Prospective, randomized, partially masked study	TES	DTL electrode	Biphasic intensity 150% or 200% of EPT, 5 ms/phase, 20 Hz, 30 min, once a week for 1 yr	Reduction of loss of VF
Miura et al. (2023)	ION (n = 5)	Prospective, non-randomized, open-label, uncontrolled study	TdES	Skin electrode (lower eye lid)	Biphasic 1.0 mA, 10 ms/phase, 20 Hz, 30 min, every 2 wk for 6 sessions	Improvement in VA or VF

BRAO, branch retinal artery occlusion; BVMD, Best vitelliform macular dystrophy; CRAO, central retinal artery occlusion; DTL, Dawson–Trick–Litzkow; ERG, electroretinogram; ETDRS, Early Treatment Diabetic Retinopathy Study; EPT, electrical phosphene threshold ; GVF, Goldmann visual field; HFA, Humphrey field analyzer; ION, ischemic optic neuropathy; LHON, Leber hereditary optic neuropathy; mERG, multifocal electroretinogram; NTG, normal tension glaucoma; OND, optic nerve damage; RP, retinitis pigmentosa; rtACS, transorbital alternating current stimulation; TdES, transdermal electrical stimulation; TES, transcorneal electrical stimulation; TON, traumatic optic neuropathy; qCSF, quick contrast sensitivity function; VA, Visual acuity; VF, visual field.

Clinical studies on the neuroprotective effects of ES, involving many patients, have been conducted for both RP and optic neuropathies. Among these, Schatz et al. (2011) conducted the first randomized controlled trial (RCT) of TES in patients with RP. The study reported the safety of TES in RP patients. and enhancements in the VF area (VFA) and scotopic b-wave amplitude. A continuation of this study revealed a trend toward improved safety and function (specifically scotopic b-wave amplitude) with 1 year of continued treatment (Schatz et al., 2017). Furthermore, regular and dose-dependent use of TES significantly reduced the loss of VFA (V4e) in treated eyes compared with untreated eyes in patients with RP (Stett et al., 2023).

Since then, more RCTs on TES for patients with RP have been conducted. Wagner et al. (2017) demonstrated that TES was safe and well-tolerated in patients with RP. However, visual function measurements at 6 months were not significantly different between the control and treated eyes.

With TES, some patients experienced a significant improvement in VA and VF (Bittner and Seger, 2018b). A single-arm open-label interventional trial involving 105 patients with RP reported an excellent safety profile for TES; however, it did not observe significant improvements in visual function (Jolly et al., 2020). Another single-arm open-label interventional trial with 101 patients with RP found that the mean BCVA and VF test scores improved significantly 1 month after TES initiation. However, these improvements were transient and disappeared after the treatment was discontinued (Sinim Kahraman and Oner, 2020). In addition, single-arm open-label interventional trials have suggested that TES may slow deterioration in multifocal electroretinography (Demir et al., 2022; Dizdar Yigit et al., 2022). Furthermore, RCTs investigating transdermal ES (TdES), a technique similar to TES, in patients with RP demonstrated both safety and significant improvements in VA and VF for up to 3 months (Miura et al., 2019).

In clinical applications of TES and TdES for RP, some patients demonstrate notable improvements in visual function, whereas others do not exhibit significant changes. Such heterogeneity in outcomes is likely attributable to multiple factors, including disease stage and severity, inter-individual anatomical and physiological differences, and variations in stimulation parameters (e.g., current intensity, frequency, duration, and interval of treatment sessions).”

TES has shown potential in improving VA and VF in patients with RP. However, as the underlying disease continues to progress, these improvements may be temporary, eventually giving way to further deterioration in visual acuity and visual field. Therefore, a critical challenge in clinical practice is to develop strategies that can sustain the therapeutic effects of TES and help slow the progression of retinal degeneration over time.

TES has been widely performed for patients with various optic neuropathies. An RCT of repetitive transorbital alternating current stimulation (rtACS), which is similar to TES, for patients with ON damage, such as traumatic optic neuropathy, revealed that rtACS facilitated vision restoration in VA and VF size (Sabel et al., 2011; Gall et al., 2011; Fedorov et al., 2011; Gall et al., 2016).

Preliminary studies have investigated the potential of TdES as a treatment option for optic neuropathies. In a study on Leber hereditary optic neuropathy, 10 patients received TdES over 10 weeks. Significant improvements in VA were observed at all follow-up points, with half of the patients demonstrating notable enhancements in VF sensitivity (Kurimoto et al., 2020). Another study evaluated TdES for nonarteritic anterior ischemic optic neuropathy in five patients treated over 12 weeks. Some cases showed improvements in VA and VF sensitivity without adverse events (Miura et al., 2023).

Despite the relatively few treatment reports on the effects of TES on optic neuropathies (Fujikado et al., 2006), results of previous animal experiments and clinical trials of rtACS suggest the significant potential efficacy of TES for these conditions. Therefore, RCTs are needed to further investigate the therapeutic effects of TES on optic neuropathies.

Finally, regarding the safety of TES treatment, numerous studies to date have reported no serious complications, with only transient dry eye symptoms and punctate superficial keratitis being observed (Jolly et al., 2020; Sinim Kahraman and Oner, 2020). TES induces corneal epithelial damage in mice by disrupting mucin homeostasis (Yang et al., 2022). However, these were mild and all resolved without sequelae (Jolly et al., 2020; Sinim Kahraman and Oner, 2020).

Although potential side effects of TdES treatment—such as keratitis, dermatitis, facial or trigeminal nerve disorders, and nasal abnormalities—were anticipated due to the use of skin electrodes, none of these adverse events were observed during treatment. The skin sensory irritation and discomfort caused by the electrical stimulation were well tolerated by the patients (Kurimoto et al., 2020; Miura et al., 2023).

## 7 Clinical potential of TES for treating brain disorders

As mentioned earlier, TES stimulates the retina, resulting in phosphene generation in the visual cortex of the brain. Thus, ES of the eye affects the CNS. Because the eyes are an extension of the brain, examining ocular symptoms is gradually becoming a common practice in diagnosing brain pathologies.

Ophthalmological evaluations have revealed that neurodegenerative and neurological diseases, such as AD, PD, and multiple sclerosis, manifest retinal symptoms (Majeed et al., 2021; Chang et al., 2022; Bostan et al., 2023).

To treat these diseases, interest is growing in leveraging the connection between the eyes and the brain for therapeutic interventions. Most forms of ES of the brain are invasive, such as deep brain stimulation and motor cortex stimulation, and often involve postoperative complications. Conversely, noninvasive forms, such as tES and VNS, exhibit significant variability in response to the stimulation (Reed and Cohen Kadosh, 2018).

TES is considered a novel approach for noninvasively stimulating the eye to modulate brain networks in neurodegenerative diseases. TES to the retina modulates the brain coherence and connectivity of the visual and nonvisual cortices, and the observed alterations are largely maintained. TES holds a strong potential to modulate higher cortical functions, including cognition, awareness, emotion, and memory (Agadagba et al., 2022).

In rat models of retinal degeneration and chronic unpredictable stress, TES has shown promising effects, such as promoting antidepressant-like actions and recovering cognitive impairments (Yu et al., 2021; Yu et al., 2022a; Yu et al., 2022b). Despite the lack of basic or clinical research on the therapeutic effects of TES on AD or PD, noninvasive TES shows potential as a tool for modulating brain function in the treatment of brain diseases. This approach offers hope for the future treatment of patients with neurodegenerative diseases.

## 8 Concluding remarks and future directions

This review summarizes the results of studies on the role of TES as an assessment method of inner retinal layer function in patients with photoreceptor degeneration and as a neuroprotective treatment for retinal and ON diseases. Furthermore, it explores foundational research investigating the potential of TES as a neuroprotective therapy for brain disorders. TES not only influences retinal function by promoting neurotrophic factor production and immunosuppression and increasing blood flow but also affects other brain regions, including the visual cortex and hippocampus. With further clinical advancements, TES shows promise as a therapeutic approach for degenerative conditions of the retina and ON and neurological disorders.
